# Results of a Single Institution Experience with Dose-Escalated Chemoradiation for Locally Advanced Unresectable Non-Small Cell Lung Cancer

**DOI:** 10.3389/fonc.2017.00001

**Published:** 2017-01-23

**Authors:** Mark E. Bernard, Scott M. Glaser, Beant S. Gill, Sushil Beriwal, Dwight E. Heron, James D. Luketich, David M. Friedland, Mark A. Socinski, Joel S. Greenberger

**Affiliations:** ^1^Department of Radiation Oncology, University of Pittsburgh Cancer Institute, Pittsburgh, PA, USA; ^2^Department of Thoracic Surgery, University of Pittsburgh Medical Center, Pittsburgh, PA, USA; ^3^Division of Hematology/Oncology, Department of Medicine, University of Pittsburgh Medical Center, Pittsburgh, PA, USA

**Keywords:** NSCLC, dose escalation, chemoradiation, esophagitis, pneumonitis

## Abstract

**Background:**

We determined factors associated with morbidity and outcomes of a series of non-small cell lung cancer (NSCLC) patients treated with dose-escalated chemoradiotherapy at the University of Pittsburgh Lung Cancer Program.

**Methods and materials:**

The records of 170 stage III NSCLC patients treated with definitive intent were retrospectively reviewed. All patients received four-dimensional CT simulation scan and had respiratory gating if tumor movement exceeded 5 mm. Overall survival (OS), locoregional control (LRC), and freedom from distant metastasis (FFDM) were calculated using log-rank and Cox regression analysis.

**Results:**

For the present series of patients, median follow-up was 36.6 months, median survival 27.4 months, and the 2- and 4-year OS was 56.0 and 30.7%, respectively. The 4-year LRC and FFDM were 43.9 and 40.7%, respectively. No benefit was associated with irradiation doses above 66 Gy in OS (*p* = 0.586), LRC (*p* = 0.440), or FFDM (*p* = 0.230). On univariate analysis, variables associated with worse survival included: clinical stage IIIB (*p* = 0.037), planning target volume (PTV) over 450 cc (*p* < 0.001), heart V_30_ over 40% (*p* = −0.048), and esophageal mean dose over 20% (*p* = 0.024), V_5_ (*p* = −0.015), and V_60_ (*p* = −0.011). On multivariable analysis, PTV above 450 cc (52.2 vs. 25.3 months, *p* < 0.001) and esophageal V_60_ >20% (43.8 vs. 21.3 months, *p* = −0.01) were associated with lower survival. Grade 2 or higher acute lung toxicity and esophagitis were detected in 9.5 and 59.7%, respectively of patients. Grade 2 or higher acute lung toxicity was reduced if lung V_5_ was ≤65 (7.4 vs. 23.8%, *p* = 0.03). Grade 2 or higher acute esophagitis was reduced if V_60_ ≤ 20% (62 vs. 81.3%, *p* = 0.018). The use of intensity-modulated radiation therapy was more frequent in stage IIIB compared to stage IIIA patients (56.5 vs. 39.5%, *p* = 0.048) and was associated with a higher lung V_5_ and V_10_.

**Conclusion:**

The outcomes of a program of dose-escalated chemoradiotherapy for unresectable stage IIIA and IIIB NSCLC patients were consistent with other studies and showed no benefit to radiation doses above 66 Gy. Furthermore, maintaining low esophageal V_60_ and lung V_5_ were associated with lower morbidity and mortality.

## Introduction

The optimal chemoradiation therapy management of locally advanced non-small cell lung cancer (NSCLC) has been a subject of great interest (1). Even with current aggressive multi-modality treatment protocols, the clinical outcomes remain suboptimal (2). Previous clinical trials have established the superiority of concurrent chemoradiation using radiation doses between 60 and 66 Gy and revealed median survival times of 16–18 months (3–5). Dose-escalation protocols in Phase II clinical trials revealed the feasibility of radiation doses up to 74 Gy and suggested some increase in median survival to 26 months (6). Advances in radiation therapy techniques such as intensity-modulated radiation therapy (IMRT), three-dimensional conformal radiation therapy (3D-CRT), motion management, and attention to normal tissue dose parameters allowed dose escalation in some studies (7, 8). Radiation dose-escalation trials were evaluated for improved locoregional control (LRC) and overall survival (OS).

A recent clinical Trial from the RTOG (RTOG 0617) for stage IIIA/B NSCLC (2) was initiated to randomize radiation dose groups to cohorts of 60 Gy compared to 74 Gy each with similar concurrent chemotherapy. The 74-Gy arm showed no benefit and was associated with a survival detriment. Both arms had the same LRC, distant metastasis rate and measured toxicity; however, further analysis showed that the use of IMRT was associated with better patient reported quality of life, and also that improved baseline pretreatment QOL was predictive of survival (9). Other parameters influencing survival included low heart dose, low esophageal morbidity, and smaller planning target volume (PTV). Although, it is important to note, the lack of pretreatment quality assurance from a central review has called the outcomes in question, especially when it comes to delineating normal structures such as the heart (10, 11).

We now report the results of this single institution experience with definitive chemoradiation for stage IIIA and IIIB NSCLC and compare outcomes for patients treated with 3D-CRT compared to patients treated with IMRT.

## Materials and Methods

### Patient Population

We carried out a retrospective study using the criteria established by the American Joint Committee on Cancer (AJCC) seventh edition for patients with Stage IIIA and IIIB NSCLC. All patients were treated with definitive intent over the interval 2001–2013. All patients were determined to be unresectable by either: multiple node positivity, contralateral or supraclavicular lymph nodes, or tumor invasion of adjacent organs.

Patient medical records were de-identified and analyzed with ethical approval by the University of Pittsburgh Institutional Review Board (IRB #PRO13020306). The review was done with compliance to our ethic standards. All patients received daily radiotherapy fractions using a megavoltage linear accelerator with photon energy above 6 MV, and either IMRT or 3D-CRT. Radiation therapy was delivered concurrently with dual agent chemotherapy. All patients received four-dimensional CT (4D-CT) scan to assess target motion at time of simulation. Respiratory gating was included in daily treatments if tumor movement during simulation was greater than 5 mm in any direction (12).

### Staging

The staging system for NSCLC changed during the years of the study, therefore, we used the AJCC seventh staging edition as a standard for all patients. Patients were staged using PET/CT of the chest, abdomen, and pelvis, brain MRI, mediastinal lymph node sampling, and biopsy of both primary cancer and multiple mediastinal lymph nodes.

### Dosimetry Parameters

In general, the normal esophagus was defined as being from the cricopharyngeus muscle to the gastroesophageal junction and the lung volume including the bilateral lungs from the apex chest apex to the diaphragm while subtracting out the PTV. These were mostly already created using our treatment plan software and we also used paper charts to assist with determine dosimetry values. In general our goals were V20 for lung <30%, mean esophagus dose less than or equal to 35 Gy and cord dose less than or equal to 45 Gy. Heart dose varied based on tumor location. For IMRT we also added V5 <60% and V10 <40% constraints for lung. In our early experience, patients were treated with fixed-field IMRT and after expanded to both rapid arc and fixed-field IMRT.

### Statistical Analysis

LRC, freedom from distant metastasis (FFDM), and OS were primary outcome measures, and were calculated using log-rank and Cox regression analysis from time of diagnosis. Locoregional control was defined as lack of progression of clinical disease, seen on follow-up imaging or biopsy of the radiation therapy treatment volume for primary and regional lymph nodes. Acute toxicity was evaluated using binomial regression and late toxicity evaluated using Cox regression, *t*-test, ANOVA, binomial regression, and linear regression analysis. Each outcome measurement was correlated to dosimetric variables. Multivariable analysis (MVA) was performed for each factor found to be significant on unvariant analysis (*p* ≤ 0.05). Statistical analysis was performed using IBM SPSS Statistics Version 23.

## Results

### Patient Demographics

There were 597 patients with clinical stage IIIA or IIIB NSCLC in the UPCI database over the interval 2001 to 2013. Patients treated with palliative intent (*n* = 296) were excluded as were those receiving only surgical management (*n* = 28). Radiotherapy patients not treated with concurrent chemotherapy (*n* = 39) were excluded. The remaining cohort of 213 patients was evaluated. Forty-three patients were lost to follow-up defined as no follow-up from treatment. Median follow-up of the cohort of 170 patients was 22 months and is shown in Table 1. The Interquartile Range (IQR) was 8.9–39.6. Median follow-up was 36.6 months (IQR, 26.6–63.7) for the subset of patients still living.

**Table 1:** **Baseline characteristics for entire cohort (*n* = 170)**.

Patient variables	Results
**Age**	
Median and range (years)	67 (38–91)

**Gender**	
Male	111 (65.3%)
Female	59 (34.7%)

**Race**	
Caucasian	137 (80.6%)
African-American	33 (19.4%)
NOS/other	

**Histology**	
Adenocarcinoma	77 (45.3%)
Squamous cell	54 (31.8%)
Large cell	10 (5.9%)
Mixed	2 (1.2%)
NSCLC/NOS	27 (15.9%)

**Stage**	
T4N0	10 (5.9%)
T3N1	3 (1.8%)
T4N1	3 (1.8%)
T0N2	8 (4.7%)
T1N2	21 (12.4%)
T2N2	36 (21.2%)
T3N2	16 (9.4%)
T4N2	20 (11.8%)
T0N3	8 (4.7%)
T1N3	11 (6.5%)
T2N3	17 (10%)
T3N3	7 (4.1%)
T4N3	10 (5.9%)

**Stage**	
IIIA	97 (57.1%)
IIIB	73 (42.9%)

**Chemotherapy**	
Carboplatin paclitaxel	150 (88.2%)
Cisplatin and etoposide	5 (2.9%)
Carboplatin and protein-bound paclitaxel	1 (0.6%)
Cisplatin and gemcitabine	1 (0.6%)
Carboplatin and pemetrexed	2 (1.2%)
Carboplatin and etoposide	5 (2.9%)
Carboplatin and docetaxel	3 (1.8%)
Cisplatin and docetaxel	3 (1.8%)

**Volumes**	
Median GTV (cc)	84 (4–586)
Median PTV (cc)	338 (43–1303)

**Technique**	
3D-RT	119 (70%)
IMRT	46 (27.1%)
N/A	5 (2.9%)

**Simulation**	
No gating	127 (74.7%)
Gating	23 (13.5%)
N/A	20 (11.8%)

**Dose (Gy)**	
Median and range	72 (54–84)

**Dose ranges (Gy)**	
54–66	41 (24.1%)
67–70	32 (18.8%)
71–74	51 (30%)
75–80	45 (26.5%)
81–84	1 (0.6%)

*GTV, gross tumor volume; NA, not available; NSCLC, non-small cell lung cancer; NOS, not otherwise specified; 3D-CRT, three-dimensional conformal radiation therapy; IMRT, intensity-modulated radiation therapy; PTV, planning target volume*.

### Treatment and Dosimetric Evaluation

All patients were treated with concurrent chemoradiation, median radiation dose was 72 Gy (IQR, 68–77). The treatment volume and doses had inhomogeneity corrections. IMRT was used in 27.1%. All patients received a 4D-CT simulation scan, and 13.5% required respiratory gating. Concurrent carboplatin and paclitaxel were the dual agents in 88.2% of patients.

### Overall Survival

The median survival of the entire group was 27.4 months. There was a 2- and 4-year OS of 56.0 and 30.7%, respectively. On univariate analysis decreased survival was detected in the subset of patients with: (1) stage IIIB (*p* = 0.037), (2) PTV > 450 cc (*p* < 0.001), (3) heart V_30_ > 40% (*p* = 0.048), (4) esophageal mean dose of >20% (*p* = 0.024), (5) esophageal V_5_ > 60% (*p* = 0.015), and (6) esophageal V_60_ > 20% (*p* = 0.011). On multivariate analysis decreased survival was detected in patients with a large PTV (≥450 vs. <450 cc; 52.2 vs. 25.3 months, *p* < 0.001). Esophageal volumes of V_60_ that were over 20% were also associated with reduced survival (V_60_ ≤ 20 vs. >20; 43.8 vs. 21.3 months, *p* = 0.01). Radiation dose above 66 Gy was not associated with improved OS (*p* = 0.586). Statistical analysis is shown in Table 2.

**Table 2:** **Univariate and multivariable analysis (MVA) for overall survival (OS), LRC, and freedom from distant metastasis (FFDM)**.

	OS	Locoregional control	FFDM
Age ≤60	*p* = 0.373	HR = 0.614 (0.368–1.024), *p* = 0.062	*p* = 0.261
Gender	*p* = 0.166	*p* = 0.408	*p* = 0.686
Race	*p* = 0.742	*p* = 0.540	*p* = 0.926
Histology	*p* = 0.155	*p* = 0.904	*p* = 0.562
IIIA vs. IIIB	**HR = 1.465; 95% confidence interval (CI) (1.024–2.096), *p* = 0.037**	*p* = 0.467	**HR = 0.598, 95% CI (0.374–0.956), *p* = 0.032**
RT dose (continuous)	*p* = 0.761	*p* = 0.706	0.511
≤66 vs. >66 Gy	*p* = 0.586	*p* = 0.440	*p* = 0.230
3D vs. IMRT	*p* = 0.427	*p* = 0.991	0.964
PTV_450cc_	**HR = 2.305, 95% CI (1.478–3.596), *p* < 0.001**	**HR = 1.860; 95% CI (1.031–3.356), *p* = 0.039**	**HR = 2.149, 95% CI (1.274–3.624), *p* = 0.004**

**Lung**			
Mean	*p* = 0.402	–	–
V_5_	*p* = 0.584	–	–
V_10_	*p* = 0.519	–	–
V_20_	*p* = 0.474	–	–
V_30_	*p* = 0.186	–	–

**Heart**			
Maximum	*p* = 0.242	–	–
Mean	*p* = 0.091	–	–
V_30_ ≤ 40	**HR = 1.836, 95% CI (1.005–3.353), *p* = 0.048**	–	–

**Esophageal**			
Max	*p* = 0.408	–	–
Mean ≤ 20	**HR = 2.146, 95% CI (1.105–4.167), *p* = 0.024**	–	–
V_5_ ≤ 60	**HR = 1.017, 95% CI (1.003–1.030), *p* = 0.015**	–	–
V_60_ ≤ 20	**HR = 1.758, 95% CI (1.135–2.721), *p* = 0.011**	–	–

**MVA**			
PTV_450cc_	**52.2 vs. 25.3 months, *p* < 0.001**	**HR = 1.860; 95% CI (1.031–3.356), *p* = 0.039**	**HR = 2.149, 95% CI (1.274–3.624), *p* = 0.004**
Esophageal V_60_ ≤ 20	**43.8 vs. 21.3 months, *p* = 0.01**	–	–

*3D-CRT, three-dimensional conformal radiation therapy; IMRT, intensity-modulated radiation therapy; PTV, planning target volume; RT, radiation therapy*.

### Locoregional Control

The 2-and 4-year LRC for the entire group was 54.4 and 43.9%, respectively. On univariate analysis, large PTV was associated with a reduced LRC [≤450 vs. >450 cc, HR = 1.86; 95% confidence interval (CI) (1.03–3.36), *p* = 0.039]. Radiation dose above 66 Gy was not associated with increased LRC (*p* = 0.440).

### Freedom from Distant Metastasis

The percentage of 2- and 4-year FFDM was 54.4 and 40.7%, respectively, for the entire group. On univariate analysis, higher radiation therapy doses (*p* = 0.041) and PTV > 450 cc (*p* = 0.004) were each associated with reduced FFDM. On multivariate analysis, PTV above 450 cc [≤450 vs. >450 cc, HR = 2.15, 95% CI (1.27–3.62), *p* = 0.004] was associated with reduced FFDM. A radiation dose above 66 Gy was not associated with an improved FFDM (*p* = 0.230).

### Factors Influencing Toxicity

In the present series, the median value of mean lung dose was 15.2 Gy (IQR, 13–18 Gy) and median lung V_20_ was 26% (IQR 21–31%). There was grade 2 or higher acute lung toxicity detected in 9.5% of patients. On univariate analysis, the use of 3D-CRT (*p* = 0.036) and V_5_ ≤ 65 (*p* = 0.036) were associated with a lower rate of pneumonitis. On multivariate analysis, lung V_5_ ≤ 65 was associated with a decreased acute grade 2 or higher lung toxicity (7.4 vs. 23.8%, *p* = 0.027). The incidence of 1-year and 2-year late grade 3+ pneumonitis was 1.2 and 2.1%, respectively. There were no detectable univariate factors that predicted for late grade 3 or higher pneumonitis.

The median value for the mean esophageal dose in the group was 28 Gy in the present study (IQR, 21–33 Gy). The median esophageal V_60_ was 16% (IQR, 3–27%). Acute grade 2 or higher esophagitis was detected 59.7% of patients. On univariate analysis, a lower radiation therapy dose as a continuous variable (*p* = −0.041) and esophageal V_60_ ≤ 20% (*p* = −0.027) were associated with lower rates of acute grade 2 or higher esophagitis. On multivariate analysis, esophageal V_60_ ≤ 20% was associated with a lower rate of acute grade 2 or higher esophagitis (62 vs. 81.3%, *p* = 0.018). We observed late 1-year and 2-year grade 3+ esophagitis in 4.5 and 6.5% of patients, respectively. There was no detectable univariate factor that predicted late grade 3 or higher esophagitis (Table 3).

**Table 3:** **Univariate and multivariable analysis (MVA) for acute toxicity**.

	Acute grade 2+ lung toxicity	Acute 2+ esophagitis
**3D-CRT** vs. **IMRT**	**HR = 3.181 (1.079–9.384), *p* = 0.036**	*p* = 0.086
**RT dose**	*p* = 0.626	**HR = 1.060, 95% confidence Interval (CI) (1.002–1.120, *p* = 0.041)**
**PTV**	*p* = 0.720	*p* = 0.951

**Lung**		
Mean	*p* = 0.893	–
V_5_ ≤ 65	**HR = 3.884, 95% CI (1.096–13.767), *p* = 0.036**	–
V_10_	*p* = 0.377	–
V_20_	*p* = 0.345	–
V_30_	*p* = 0.895	–

**Esophagus**		
Max	–	0.743
Mean	–	0.077
V_5_	–	0.208
V_60_ ≤ 20	–	**HR = 2.2697, 95% CI (1.115–6.340), *p* = 0.027**

**MVA**		
V_5_ ≤ 65	**7.4 vs. 23.8%, *p* = 0.027**	**–**
Esophageal V_60_ ≤ 20		**62 vs. 81.3%, *p* = 0.018**

*3D-CRT, three-dimensional conformal radiation therapy; IMRT, intensity-modulated radiation therapy; PTV, planning target volume, RT, radiation therapy*.

### Utilization and Outcomes of 3D-CRT vs. IMRT

We compared factors that were associated with the radiation oncologist’s choice of 3D-CRT vs. IMRT for radiotherapy management. We found no detectable difference by patient age (*p* = 0.095), gender (*p* = 0.072), race (*p* = 0.340), histology (*p* = 0.752), RT dose (*p* = 0.131), respiratory gating (*p* = 0.105), or PTV (*p* = 0.459). IMRT use was higher in IIIB patients than in IIIA patients (56.5 vs. 39.5%, *p* = 0.048). For IIIB compared to IIIA patients, IMRT was associated with a higher lung V_5_ (37 vs. 57%, *p* = 0.001) and V_10_ (30 vs. 43%, *p* = 0.002). Compared to patients treated with 3D-CRT, there was a lower esophageal mean dose for IMRT patients (69 vs. 74%, *p* = 0.049) (Table 4). The median survival for IIIB patients treated with IMRT was higher than those treated with 3D-CRT, but the data did not reach statistical significance (19.0 vs. 26.1 months, *p* = 0.429). IMRT use was also associated with improved survival for IIIA patients, but did not reach statistical significance (28.7 vs. 42.3 months, *p* = 0.418).

**Table 4:** **Dosimetric outcomes for 3D-CRT vs. intensity-modulated radiation therapy (IMRT) in IIIA and IIIB non-small cell lung cancer patients**.

		Mean	*p*-Value
**Clinical IIIA**			

**Lung**			
Mean	3D	15	0.491
	IMRT	16	
V_5_	3D	37	0.001
	IMRT	57	
V_10_	3D	30	0.002
	IMRT	43	
V_20_	3D	23	0.195
	IMRT	26	
V_30_	3D	19	0.758
	IMRT	19	

**Heart**			
Maximum	3D	56	0.068
	IMRT	68	
Mean	3D	12	0.430
	IMRT	14	
V_30_	3D	15	0.716
	IMRT	17	

**Esophagus**			
Max	3D	69	0.049
	IMRT	74	
Mean	3D	25	0.528
	IMRT	27	
V_5_	3D	57	0.288
	IMRT	61	
V_60_	3D	14	0.785
	IMRT	15	

**Clinical IIIB**			

**Lung**			
Mean	3D	17	0.774
	IMRT	17	
V_5_	3D	45	0.009
	IMRT	59	
V_10_	3D	33	0.004
	IMRT	45	
V_20_	3D	28	0.413
	IMRT	30	
V_30_	3D	23	0.240
	IMRT	21	

**Heart**			
Maximum	3D	71	0.026
	IMRT	53	
Mean	3D	17	0.133
	IMRT	12	
V_30_	3D	24	0.054
	IMRT	13	

**Esophagus**			
Max	3D	74	0.452
	IMRT	72	
Mean	3D	34	0.739
	IMRT	33	
V_5_	3D	66	0.087

## Discussion

Despite advances in delivery techniques for radiation therapy, patients with locally advanced NSCLC have displayed suboptimal outcomes, and median survival remains between 15 and 26 months (2, 13). Dose-escalation protocols for radiation therapy doses above 60 Gy showed a benefit, but also revealed increased toxicities (14). The addition of concurrent chemotherapy to 60 Gy radiation resulted in further improved survival (3–5). While some phase II studies showed the feasibility of dose-escalated chemoradiation therapy with higher survival outcomes, the recent RTOG 0617 study did not confirm a benefit to the use of higher radiation dose (2, 6). We compared the RTOG 0617 results with those of our single institution series at UPCI.

The median survival for the present cohort of 170 evaluable NSCLC stage IIIA and IIIB patients was 27.4 months. This value is comparable to the median survival reported in the recent RTOG 0617 trial. In both studies, better staging with PET/CT leading to stage migration and improvements in treatment delivery and better supportive care may have accounted for the improvement in survival (2).

The median radiation dose delivered to patients in our single institution study was 72 Gy. We determined the value of dose escalation by comparing those patients treated to doses above 66 Gy with those receiving doses below 66 Gy. This cut-off value was based upon prior clinical trials comparing sequential to concurrent chemoradiation (3–5). Prior trials showed that concurrent, dual agent, platinum-based chemotherapy was more effective than use of sequential single drugs in chemoradiation protocols (3–5). We found no benefit for those patients treated above 66 Gy. We also found no benefit if the radiation dose was analyzed as a continuous variable. Therefore, we conclude that our data correlate with the results of RTOG 0617 showing no benefit to radiation dose escalation above 66 Gy.

The present analysis revealed that PTV volumes of NSCLC tumor above 450 cc were associated with a lower OS, LRC, and FFDM, again confirming the results presented in RTOG 0617 (2). Another clinical trial, which is a randomized phase II program, which evaluates adaptive planning in stage IIIA/B NSCLC patients for dose escalation, RTOG 1106, is ongoing. The control arm in this new study will receive 50 Gy, then continuing to 60 Gy after an interim PET/CT scan. The experimental arm will receive 46.2 Gy, then PET/CT scan evaluation, then use adaptive dose-escalation ranges using the FDG-PET/CT scan up to a total dose of 80.4 Gy.

Our study revealed new information on toxicity of chemoradiotherapy of NSCLC. Acute grade 2 or higher lung toxicity in 9.5% patients was associated with a median lung V_20_ of 26 Gy. The data correlate well with a prior study showing that V_20_ values between 22 and 31 Gy led to an 8% pneumonitis rate (8). In this prior study (8), 42% of patients received chemotherapy (concurrent or sequential). In our study, the low rate of pneumonitis with high dose concurrent chemoradiation was likely attributable to 4D-CT simulation and the pre-screening of patients with tumor movement to include respiratory gating in their treatment program. Lower rates of lung toxicity were also detected in patients, where there was V_5_ ≤ 65%. A prior study (15), showed that V_5_ ≤ 42% did indeed correlate with a lower rate of acute pneumonitis for NSCLC patients treated with concurrent chemoradiation (15). A review of 220 esophageal cancer patients (16) also treated with radiation alone showed that V_5_ ≤ 60% correlated with lower rates of acute pneumonitis. We conclude that mean lung dose and lung V_20_ are the standard dosimetric parameters for predicting pneumonitis; however, we suggest that more attention should be given to the low dose volumes when treating patients with IMRT.

Esophagitis remains a serious complication of chemoradiotherapy of NSCLC. Esophageal grade 2 or higher toxicity rate was observed in 59.7% of our patients. This result was consistent with the combined grade 2 or higher esophagitis rate of the 74 Gy arm (43%) in the RTOG 0617 study (2). Keeping the esophageal V_60_ ≤ 20% correlated with lower frequency of detection of acute grade 2 or higher esophagitis. A retrospective analysis of another series of 109 NSCLC patients treated with concurrent chemoradiation showed the esophageal V_60_ correlated with higher rates of acute esophagitis (17). In yet another study, the threshold dose of 58 Gy predicted acute grade 3 or higher esophageal toxicity (18). Therefore, there is a consensus that the magnitude of the esophageal volume receiving more than 40–50 Gy correlates with the severity of acute esophagitis (19, 20). Our results support efforts to ensure that as much of the esophagus as possible should be spared from the high-dose region. We will plan on incorporating our knowledge of the esophageal V_60_ this into our treatment planning for stage III patients treated with definitive chemoradiation.

The RTOG 0617 study showed that the use of IMRT did improve the QOL in NSCLC patients and reduced rates of grade 3 pneumonitis. In addition, there was an increased likelihood of completing adjuvant chemotherapy (2, 9). We found that IMRT use was greater for IIIB patients, likely due to the requirement for coverage of supraclavicular and contralateral mediastinal disease, while achieving acceptable V_20_ volumes (mean, 30%) and attempting to still reduce lung toxicity. IMRT treatment plans decreased the esophageal V_60_ volume and the maximum heart dose. There was a trend for IMRT treatment plans to decrease the heart V_30_ volume, a parameter associated with survival outcomes on univariate analysis, but not multivariate analysis. A lowered heart V_30_ was also associated with improved survival outcomes in the RTOG 0617 study (2).

There was no statistically significant increase in survival for IIIA or IIIB patients treated with IMRT vs. 3D-CRT, although median survival times were different. In a secondary analysis of the data in RTOG 0617, the high-dose arm was associated with a lower patient reported QOL at 3 months, and baseline pretreatment QOL was predictive of survival (9). IMRT use was associated with a less deterioration of QOL compared to patients receiving 3D-CRT. We were unable to associate use of IMRT with a possible survival benefit, although a trend in this direction was apparent. A larger study with a more balanced distribution between uses of IMRT compared to 3D-RT may result in a statistically significant difference.

Patients treated with IMRT did not show a decreased rate of acute esophagitis or pneumonitis. This result may have been attributable to a higher usage of IMRT for patients with IIIB disease, and the need for larger treatment volumes. Two retrospective reviews of NSCLC patients showed decreased rates of pneumonitis with use of IMRT when compared to 3D-CRT for NSCLC (7, 21). Radiation therapy planning comparing 3D-CRT to IMRT showed that IMRT also lead to a 50% relative reduction in predicted esophageal complications (22).

**Figure 1:**
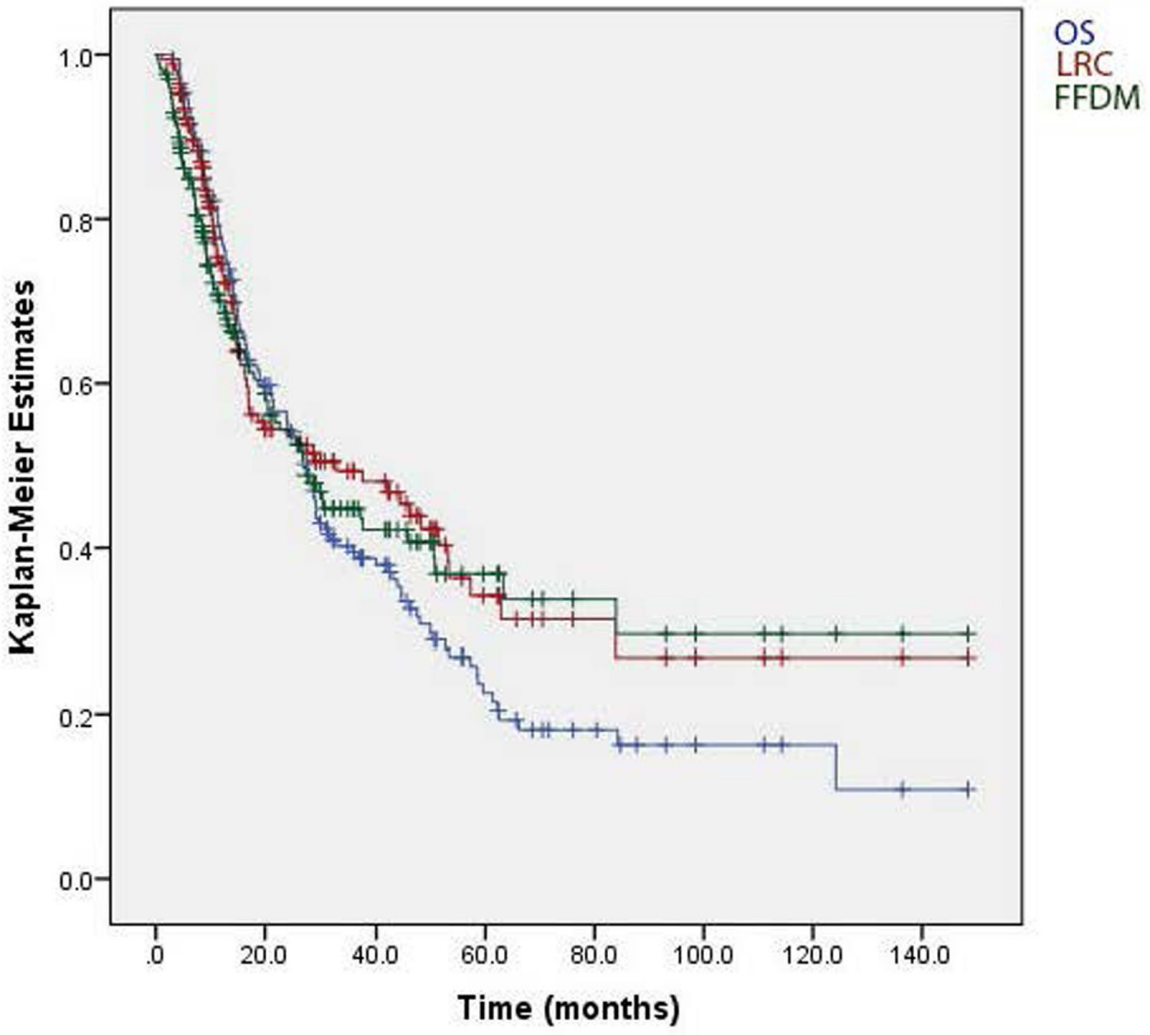
**Comparison of the overall survival (OS), locoregional control (LRC), and freedom from distant metastasis (FFDM) for stage IIIA/B non-small cell lung cancer**. The median survival was 25.9 months for the entire cohort. Both local recurrence and distant metastasis remained a major factor decreasing survival.

We analyzed our data using Kaplan–Meier plots, and this method revealed separation of the LRC and FFDM curves from OS (Figure 1). The data suggest that patients in our cohort may have died from causes other than local or distant recurrence of NSCLC. Since patients with aerodigestive cancers have a 3–5% yearly risk of a second aerodigestive primary cancer, we recommend that radiation oncologists ensure that long-term follow-up visits include screening for second cancers.

There were limitations to our retrospective analysis of NSCLC patients, including: (1) single institution retrospective review, (2) lack of a prospective randomized trial, and (3) limited follow-up interval. These factors might have led to both overestimating the primary outcomes and underestimating late toxicity. We also have a limited number of patients, and thus, our reports should be compared with other single institution reports and randomized control trials. We are additionally limited since we did not have electronic records for most of our patients and, therefore, may lead unreported events. Last, our dosimetry data are based on treating physicians who may have used different treatment considerations. We have overcome this issue by implementation of clinical pathways to guide our physicians and to have a more uniform criterion for care.

In a retrospective review of stage IIIA/B, NSCLC patients at UPCI larger PTV volumes and esophageal V_60_ > 20% were associated with poorer survival. Acute grade 2 or higher lung toxicity was lower in the V_5_ ≤ 65% group and acute grade 2 plus esophagitis was lower in the V_60_ ≤ 20% group. We recommend that patients with unresectable stage IIIA and IIIB NSCLC, who require radiotherapy be treated to doses not exceeding 66 Gy.

## Author Contributions

MB performed the retrospective review of the data analyzed in the manuscript and performed the majority of the statistics and was responsible for drafting and editing the manuscript. SG assisted with statistics and drafting of the manuscript. BG assisted in drafting the manuscript, statistical analysis, and literature review. SB oversaw drafting of the manuscript and edits and outlined the message and topic. DH assisted in drafting the manuscript and providing edits. JL provided edits concerning surgical outcomes. DF and MS reviewed the manuscript for medical oncology. JG led the team and edited the manuscript.

## Conflict of Interest Statement

The authors declare that the research was conducted in the absence of any commercial or financial relationships that could be construed as a potential conflict of interest. The reviewer PR and handling Editor declared their shared affiliation, and the handling Editor states that the process nevertheless met the standards of a fair and objective review.
